# Clinical characteristics and drug susceptibility profiles of *Mycobacterium abscessus* complex infection at a medical school in Thailand

**DOI:** 10.1186/s12941-023-00637-4

**Published:** 2023-09-21

**Authors:** Songkiat Sukmongkolchai, Suthidee Petsong, Nont Oudomying, Ajala Prommi, Sunchai Payungporn, Warat Usawakidwiree, Kanphai Wongjarit, Gompol Suwanpimolkul, Kiatichai Faksri, Chusana Suankratay, Suwatchareeporn Rotcheewaphan

**Affiliations:** 1 Medical Microbiology, Interdisciplinary and International Program, Graduate School, Chulalongkorn University, Bangkok, Thailand; 2 Department of Microbiology, Faculty of Medicine, Chulalongkorn University, Bangkok, Thailand; 3 Chulalongkorn University International Medical Program (CU-MEDi), Faculty of Medicine, Chulalongkorn University, Bangkok, Thailand; 4 Program in Bioinformatics and Computational Biology, Graduate School, Chulalongkorn University, Bangkok, Thailand; 5 Center of Excellence in Systems Microbiology, Faculty of Medicine, Chulalongkorn University, Bangkok, Thailand; 6 Department of Biochemistry, Faculty of Medicine, Chulalongkorn University, Bangkok, Thailand; 7 Department of Medicine, Faculty of Medicine, Chulalongkorn University, Bangkok, Thailand; 8 Division of Infectious Diseases, Department of Medicine, Faculty of Medicine, Chulalongkorn University, Bangkok, Thailand; 9 Department of Microbiology, Faculty of Medicine, Khon Kaen University, Khon Kaen, Thailand; 10 Research and Diagnostic Center for Emerging Infectious Diseases (RCEID), Khon Kaen University, Khon Kaen, Thailand

**Keywords:** *Mycobacterium abscessus* complex, *M. abscessus* subsp. *abscessus*, *M. abscessus* subsp. *bolletii*, *M. abscessus* subsp. *massiliense*, Drug susceptibility testing, Drug resistance, Clinical outcomes, Clinical characteristics

## Abstract

**Objectives:**

This study investigated the differences in epidemiological and clinical data, and antimicrobial susceptibilities among different subspecies of *Mycobacterium abscessus* complex (MABSC) clinical isolates at a medical school in Thailand.

**Methods:**

A total of 143 MABSC clinical isolates recovered from 74 patients were genotypically analyzed for *erm*(41), *rrl*, and *rrs* mutations, and antimicrobial susceptibilities were determined using a broth microdilution method. Patient characteristics and clinical outcomes were reviewed from the medical records.

**Results:**

Seventy-four patients were infected with 28/74 (37.8%) *M. abscessus* subspecies *abscessus* (MAB), 43/74 (58.1%) *M. abscessus* subsp. *massiliense* (MMA), and 3/74 (4.1%) *M. abscessus* subsp. *bolletii* (MBO). The clinical findings and outcomes were generally indistinguishable between the three subspecies. All three subspecies of MABSC clinical isolates exhibited high resistance rates to ciprofloxacin, doxycycline, moxifloxacin, TMP/SMX, and tobramycin. MAB had the highest resistance rates to clarithromycin (27.8%, 20/72) and amikacin (6.9%, 5/72) compared to MBO and MMA, with *p* < 0.001 and *p* = 0.004, respectively. In addition, the rough morphotype was significantly associated with resistance to amikacin (8.9%, 5/56), clarithromycin (26.8%, 15/56), and imipenem (76.8%, 43/56) (*p* < 0.001), whereas the smooth morphotype was resistant to linezolid (57.1%, 48/84) (*p* = 0.002). In addition, T28 of *erm*(41), *rrl* (A2058C/G and A2059C/G), and *rrs* (A1408G) mutations were detected in 87.4% (125/143), 16.1% (23/143), and 9.1% (13/143) of MABSC isolates, respectively.

**Conclusions:**

Three MABSC subspecies caused a variety of infections in patients with different underlying comorbidities. The drug susceptibility patterns of the recent circulating MABSC strains in Thailand were different among the three MABSC subspecies and two morphotypes.

**Supplementary Information:**

The online version contains supplementary material available at 10.1186/s12941-023-00637-4.

## Background

*Mycobacterium abscessus* complex (MABSC) is one of the most common rapidly growing mycobacteria (RGM) recovered from clinical specimens and is resistant to a variety of antimicrobial agents [1]. In Thailand, RGM, especially MABSC, have emerged as a causative agent of recalcitrant infections in patients with preexisting chronic lung diseases and people living with human immunodeficiency virus (HIV), as reported worldwide. Moreover, MABSC is associated with localized or disseminated infection in patients with adult-onset anti-interferon-gamma (IFN-γ) autoantibody syndrome, which is prevalent in Southeast and East Asia [2, 3].

MABSC can be classified into three subspecies based on their genome characteristics, namely, *M. abscessus* subsp. *abscessus* (MAB), *M. abscessus* subsp. *bolletii* (MBO), and *M. abscessus* subsp. *massiliense* (MMA) [4]. These three subspecies demonstrate different clinical manifestations and genotypic and phenotypic antimicrobial susceptibilities [5–9]. MAB and MBO harbor a functional erythromycin ribosomal methylase (41) (*erm*(41)) gene with thymine at position 28 (T28), associated with inducible clarithromycin resistance (ICR), while MMA has a truncated *erm*(41), resulting in a nonfunctional enzyme. The cytosine substitution (T28C) in MAB and MBO can decrease the ICR activity [10]. Moreover, acquired mutations in the *rrl* gene encoding the peptidyl transferase domain of 23 S ribosomal RNA at positions 2058 and 2059 (A→C/G/T) result in macrolide resistance. Acquired amikacin resistance is conferred by mutations in the 16S rRNA (*rrs*) gene, such as A1408G [11, 12]. Furthermore, the smooth and rough morphotypes of MABSC clinical isolates may differ in virulence, pathogenesis, and drug susceptibility profiles [13, 14].

This study aimed to investigate the differences in epidemiological and clinical data as well as comprehensive drug susceptibility patterns among the three subspecies of recent circulating MABSC clinical isolates in Thailand. In addition, the correlations between clinical and microbiological characteristics were determined.

## Methods

### Study population and *Mycobacterium abscessus* complex clinical isolates

MABSC clinical isolates were obtained from patients at King Chulalongkorn Memorial Hospital, Bangkok, Thailand, between January 2018 and December 2021. The patient data regarding epidemiology, clinical presentations, microbiology, and clinical outcomes were analyzed from the medical records (Table 1). Treatment outcomes of nontuberculous mycobacterial pulmonary disease (NTM-PD) were determined using the Nontuberculous Mycobacterium Network European Trials Group (NTM-NET) group consensus statement including culture conversion, microbiological cure, clinical cure, treatment failure, recurrence, relapse, reinfection, and death [15], which was modified for extrapulmonary infections but excluded culture conversion. A total of 232 MABSC clinical isolates were initially included in the study. However, 17 MABSC isolates were excluded due to bacterial contamination (n = 8), no growth after subculture (n = 3), and being mixed with other NTM (n = 5) or *M. tuberculosis* (n = 1). Finally, a total of 143 MABSC isolates, including single and sequential isolates from 74 patients diagnosed with MABSC infections according to clinical, radiological, and microbiological criteria (≥ 2 positive sputa or endotracheal aspirates with the same MABSC subspecies isolated, ≥ 1 positive BAL or sterile site) as described in the ATS/ERS/ESCMID/IDSA clinical practice guideline [16, 17], were included in this study. The Institutional Review Board (IRB) of the Faculty of Medicine, Chulalongkorn University approved this study with IRB No. 541/63 (COA No. 967/2021).

**Table 1:** Patient characteristics and *M. abscessus* complex (MABSC) clinical isolates

Characteristics	N (%) of patients				*p-value*
	Total (N = 74)	MAB (N = 28)	MBO (N = 3)	MMA (N = 43)	
Age ^(a)^, median (IQR), years	64 (49.5–70)	65.5 (48.5–69)	33 ^(b)^	64 (50–71)	0.11
Sex, Female	50 (67.6)	19 (67.9)	1 (33.3)	30 (69.8)	0.424
**Comorbidities**					
Acquired immunodeficiency	20 (27.0)	9 (32.1)	2 (66.7)	9 (20.9)	0.147
• Adult-onset IFN-γ autoantibody syndrome	8 (10.8)	4 (14.3)	1 (33.3)	3 (7.0)	0.177
• Hematologic malignancy	3 (4.1)	1 (3.6)	1 (33.3)	1 (2.3)	0.169
• Solid malignancy	8 (10.8)	3 (10.7)	0	5 (11.6)	1
• Immunosuppressive treatment	1 (1.4)	1 (3.6)	0	0	0.419
Structural lung diseases	28 (37.8)	11 (39.3)	0	17 (39.5)	0.593
• Chronic bronchiectasis	18 (24.3)	6 (21.4)	0	12 (27.9)	0.641
• TB-induced bronchiectasis	8 (10.8)	4 (14.3)	0	4 (9.3)	0.791
• NTM-PD induced chronic bronchiectasis	2 (2.7)	1 (3.6)	0	1 (2.3)	1
Procedure-related disease	7 (9.5)	1 (3.6)	0	6 (14.0)	0.433
No underlying disease identified	19 (25.7)	7 (25.0)	1 (33.3)	11 (25.6)	1
Total	74 (100.0)	28 (100.0)	3 (100.0)	43 (100.0)	0.42
**Types of infections**					
NTM pulmonary colonization	32 (43.2)	10 (35.7)	0	22 (51.2)	0.167
NTM-PD	13 (17.6)	7 (25.0)	0	6 (14.0)	0.462
Extrapulmonary disease	29 (39.2)	11 (39.3)	3 (100.0)	15 (34.9)	0.088
• Disseminated	8 (10.8)	4 (14.3)	2 (66.7)	2 (4.7)	0.014 *
• Lymphadenitis	6 (8.1)	4 (14.3)	0	2 (4.7)	0.384
• Skin and soft tissue infection	15 (20.3)	3 (10.7)	1 (33.3)	11 (25.6)	0.227
Total	74 (100.0)	28 (100.0)	3 (100.0)	43 (100.0)	0.172
**Outcomes** ^(c)^	**N (%) of patients**				***p-value***
	**Total** (N = 31)	**MAB** (N = 14)	**MBO** (N = 3)	**MMA** (N = 14)	
Clinical cure	18 (58.1)	7 (50.0)	2 (66.7)	9 (64.3)	0.869
Culture conversion	1 (3.2)	0	0	1 (7.1)	1
Died from NTM	4 (12.9)	3 (21.4)	0	1 (7.1)	0.737
Treatment failure	7 (22.6)	4 (28.6)	1 (33.3)	2 (14.3)	0.572
Died from other conditions	1 (3.2)	0	0	1 (7.1)	1
Total	31 (100.0)	14 (100.0)	3 (100.0)	14 (100.0)	0.793

* Statistically significant value

^(a)^ Age at first diagnosis or first MABSC isolated from a clinical specimen between 2018 and 2021

^(b)^ IQR could not be calculated for the MBO subspecies

^(c)^ Only patients diagnosed with NTM-PD or extrapulmonary disease with known outcomes were included in the analysis

Abbreviations: N, number; MABSC, *M. abscessus* complex; MAB, *M. abscessus* subsp. *abscessus*; MBO, *M. abscessus* subsp. *bolletii*; MMA, *M. abscessus* subsp. *massiliense*; IQR, Interquartile range; TB, Tuberculosis; NTM-PD, Nontuberculous mycobacterial pulmonary disease

### Mycobacterial isolation and culture

MABSC clinical isolates were recovered from clinical specimens (Table 2) using the sodium hydroxide-N-acetyl-L-cysteine-sodium citrate method [18]. The MABSC isolates were stored at -80 °C until use. The frozen stock of MABSC clinical isolates, *M. abscessus* ATCC19977, and *M. peregrinum* ATCC700686 were subcultured on Lowenstein-Jensen (LJ) medium at 35 °C for 3 to 5 days for the colony morphology study, drug susceptibility, and molecular testing.

**Table 2:** Clinical specimens and *M. abscessus* complex (MABSC) clinical isolates

Specimens	N (%) of isolates			
	Total (N = 143)	MAB (N = 72)	MBO (N = 7)	MMA (N = 64)
Extrapulmonary				
Blood	3 (2.1)	1 (1.4)	2 (28.6)	0
Pus/Wound aspirate	14 (9.8)	0	1 (14.3)	13 (20.3)
Tissue biopsy	25 (17.5)	13 (18.1)	3 (42.9)	9 (14.1)
Total	42 (29.4)	14 (19.4)	6 (85.7)	22 (34.4)
Pulmonary^(a)^				
BAL	23 (16.1)	9 (12.5)	0	14 (21.9)
Endotracheal aspirate	5 (3.5)	2 (2.8)	0	3 (4.7)
Sputum	73 (51.0)	47 (65.3)	1 (14.3)	25 (39.1)
Total	101 (70.6)	58 (80.6)	1 (14.3)	42 (65.6)
Grand total	143 (100.0)	72 (100.0)	7 (100.0)	64 (100.0)

^(a)^ Respiratory specimens of patients with noncystic fibrosis that met the microbiological criteria for the diagnosis of NTM-PD according to the ATS/ERS/ESCMID/IDSA guideline

Abbreviations: N, number; MABSC, *M. abscessus* complex; MAB, *M. abscessus* subsp. *abscessus*; MBO, *M. abscessus* subsp. *bolletii*; MMA, *M. abscessus* subsp. *massiliense*; BAL, Bronchoalveolar lavage

### Genotypic assays

The MABSC subspecies and mutations associated with drug resistance were analyzed using the GenoType NTM-DR assay (Hain Lifescience) [19] (n = 135) and gene sequencing (n = 8). For the GenoType NTM-DR assay, nucleic acid was extracted from mycobacterial colonies using the Genolyse kit, and further PCR amplification and reverse hybridization followed the manufacturer’s protocol.

For next-generation sequencing, genomic DNA was extracted from culture materials using the cetyltrimethylammonium bromide (CTAB)-sodium chloride extraction method [20]. Library preparations were constructed using the NEBNext® Ultra™DNA Library Prep Kit for Illumina® following the manufacturer’s protocol. NGS was carried out using a 2 × 150 paired-end (PE) configuration by the NovaSeq instrument. FastQC and Trimmomatic V0.32 were used for quality checking and trimming the sequencing reads. Adapters and low-quality sequences (Phred score < 30) were removed. The filtered reads were mapped to the *rrl*, *erm*(41), and *rrs* genes from the reference genome of *M. abscessus* ATCC19977 (Accession no.: CU458896). Single nucleotide mutations were identified using VarScan with a minimum variant allele frequency criterion of 0.01. Drug resistance prediction and variant identification were performed using the pipelines from a previous report [12].

### Drug susceptibility testing

The MICs of 16 antimicrobial agents were examined using Sensititre™ Myco RAPMYCOI (Thermo Fisher Scientific) and a broth microdilution method that were performed and interpreted according to the Clinical and Laboratory Standards Institute (CLSI) guidelines M24, Ed3 [21] and M24S [22] (Table S1). For drugs without addressed breakpoints, the MIC values were reported and calculated for the MIC_50_ and MIC_90_. Clofazimine (Sigma-Aldrich) was dissolved in dimethyl sulfoxide (DMSO) and dispensed into cation-adjusted Mueller–Hinton broth (CAMHB) using 2-fold serial dilutions (Table S1). The MIC plates were incubated at 30 ºC for 3–5 days until a positive control had sufficient growth and reexamined to observe an ICR on day 14 of incubation [21]. The colonies of *M. abscessus* ATCC19977 and *M. peregrinum* ATCC700686 were included as quality controls.

### Statistical analysis

The results are expressed as the median and interquartile range (IQR) for age, and as the number and percentage (%) for categorical variables. Data were compared using Pearson’s X^2^ or Fisher’s exact test for categorical variables. A two-sided *p*-value of < 0.05 was considered statistically significant for the analyses. Evaluation of the positive predictive value (PPV) and negative predictive value (NPV) of molecular detection of T28 was performed using a 95% confidence interval (95% CI) (*p* < 0.05). All analyses were performed using IBM SPSS version 28 statistical software.

## Results

### Patient characteristics and clinical outcomes

The clinical characteristics and MABSC subspecies of 74 patients with MABSC infection are shown in Table 1. The median age was 64 years (IQR, 49.5–70), and 50 (67.6%) were female. Most patients (81.1%) either lived or had hometowns in Central Thailand. None of the patients tested positive for HIV infection. MMA was the most common subspecies causing pulmonary and extrapulmonary infections (58.1%, 43/74), followed by MAB (37.8%, 28/74) and MBO (4.1%, 3/74). None of the patients was infected with mixed MABSC subspecies. The MABSC subspecies were not associated with any patient characteristics (n = 74) or clinical outcomes (n = 31) (*p* > 0.05), except that MBO was significantly associated with disseminated diseases (*p* = 0.014). Two of three patients with MBO infections progressed to disseminated disease due to their immunocompromised status, which was identified as adult-onset IFN-γ autoantibody syndrome (patient 3) and hematologic malignancy (Angioimmunoblastic T-cell lymphoma) postchemotherapy (patient 12) (Table S2). Patients with acquired immunodeficiency status (20/74, 27.0%) developed various infections, including pulmonary colonization (n = 8), pulmonary diseases (n = 3), skin and soft-tissue infections (SSTIs) (n = 1), lymphadenitis (n = 3), and progressed to disseminated infections (n = 5) caused by three MABSC subspecies. Structural lung diseases from noncystic fibrosis (28/74, 37.8%) were identified as risk factors for 62.2% (28/45) of patients with pulmonary diseases and colonization.

Moreover, medical procedures or exposure to natural water sources mediated SSTIs (n = 6) and lymphadenitis (n = 1) in patients without specific underlying diseases or immunocompromised status. Procedures related to SSTIs include platelet-rich plasma injection into the knee joint, multiple drug injections into the groin, unsterile acupuncture, cosmetic surgery, and postpsoas abscess surgery. One patient with dental implantation developed lymphadenitis. In addition, half of the patients (4/8, 50%) with disseminated diseases had adult-onset IFN-γ autoantibody syndrome.

In addition, the MABSC subspecies were not associated with clinical outcomes in 31 patients diagnosed with NTM-PD or extrapulmonary diseases. The correlation between clinical outcomes and morphotypes could not be determined due to the conversion or mix of morphotypes in patients with sequential MABSC isolates. Patients (n = 19) with favorable outcomes (clinical cure and culture conversion) were infected with MMA (52.6%, 10/19), MAB (36.8%, 7/19), or MBO (10.5%, 2/19) strains susceptible to clarithromycin and amikacin. However, most MAB (85.7%, 6/7) and all MBO (100.0%, 2/2) harbored an ICR. Patients diagnosed with treatment failure (n = 7) or death from MABSC infections (n = 4) were infected with a clarithromycin/amikacin-resistant strain (n = 1) or susceptible strains (n = 10), and 4/10 of them had an ICR (Table S2).

### MABSC clinical isolates and antimicrobial susceptibility patterns

MABSC clinical isolates were isolated from various types of clinical specimens. The most common extrapulmonary and pulmonary specimens were tissue biopsy (17.5%) and sputum (51.0%), respectively (Table 2). MAB and MBO were associated with pulmonary and extrapulmonary specimen types (*p* < 0.001), respectively. Last, although MMA was associated with extrapulmonary specimens, the difference was not statistically significant (*p* = 0.159). Most MABSC clinical isolates exhibited high resistance rates to ciprofloxacin (88.8%), doxycycline (91.6%), moxifloxacin (96.5%), TMP/SMX (99.3%), and tobramycin (93.7%). The partial MABSC isolates showed resistance to cefoxitin (23.8%), imipenem (56.6%), and linezolid (48.3%) (Table 3 and Figure S1). Moreover, the intermediate result rate was high for cefoxitin (74.1%) and imipenem (42.0%). The most effective drug against MABSC was amikacin (3.5%). The clarithromycin resistance of MABSC isolates increased from 14.0% (20/143) on day 3 of incubation to 42.7% (61/143) on day 14 of incubation. The MIC_50_ and MIC_90_ of amoxicillin/clavulanic acid (> 64/32 µg/mL), cefepime (> 32 µg/mL), ceftriaxone (> 64 µg/mL), and minocycline (> 8 µg/mL) were high, while clofazimine (0.5 µg/mL) and tigecycline (1 and 2 µg/mL) exhibited low values (Table S3).

**Table 3:** Summary of antimicrobial susceptibility of *M. abscessus* complex (MABSC) based on subspecies

Drugs	N (%) of isolates												*p-value*
	Total (N = 143)			MAB (N = 72)			MBO (N = 7)			MMA (N = 64)			
	S	I	R	S	I	R	S	I	R	S	I	R	
Amikacin	130 (90.9)	8 (5.6)	5 (3.5)	59 (81.9)	8 (11.1)	5 (6.9)	7 (100.0)	0	0	64 (100.0)	0	0	0.004 *
Cefoxitin	3 (2.1)	106 (74.1)	34 (23.8)	1 (1.4)	47 (65.3)	24 (33.3)	0	6 (85.7)	1 (14.3)	2 (3.1)	53 (82.8)	9 (14.1)	0.063
Ciprofloxacin	4 (2.8)	12 (8.4)	127 (88.8)	1 (1.4)	5 (6.9)	66 (91.7)	0	1 (14.3)	6 (85.7)	3 (4.7)	6 (9.4)	55 (85.9)	0.537
Clarithromycin (D3)	122 (85.3)	1 (0.7)	20 (14.0)	51 (70.8)	1 (1.4)	20 (27.8)	7 (100.0)	0	0	64 (100.0)	0	0	< 0.001 *
Doxycycline	1 (0.7)	11 (7.7)	131 (91.6)	1 (1.4)	7 (9.7)	64 (88.9)	0	0	7 (100.0)	0	4 (6.3)	60 (93.8)	0.754
Imipenem	2 (1.4)	60 (42.0)	81 (56.6)	1 (1.4)	25 (34.7)	46 (63.9)	0	3 (42.9)	4 (57.1)	1 (1.6)	32 (50.0)	31 (48.4)	0.363
Linezolid	37 (25.9)	37 (25.9)	69 (48.3)	17 (23.6)	21 (29.2)	34 (47.2)	4 (57.1)	2 (28.6)	1 (14.3)	16 (25.0)	14 (21.9)	34 (53.1)	0.227
Moxifloxacin	2 (1.4)	3 (2.1)	138 (96.5)	2 (2.8)	2 (2.8)	68 (94.4)	0	0	7 (100.0)	0	1 (1.6)	63 (98.4)	0.707
TMP/SMX	1 (0.7)	0	142 (99.3)	0	0	72 (100.0)	0	0	7 (100.0)	1 (1.6)	0	63 (98.4)	0.497
Tobramycin	1 (0.7)	8 (5.6)	134 (93.7)	1 (1.4)	7 (9.7)	64 (88.9)	0	0	7 (100.0)	0	1 (1.6)	63 (98.4)	0.172

* Statistically significant values

Abbreviations: N, number; MABSC, *M. abscessus* complex; MAB, *M. abscessus* subsp. *abscessus*; MBO, *M. abscessus* subsp. *bolletii*; MMA, *M. abscessus* subsp. *massiliense*; S, Susceptible; I, Intermediate; R, Resistant; TMP/SMX, Trimethoprim/Sulfamethoxazole.

For the MABSC subspecies, MAB was statistically significantly associated with amikacin and clarithromycin resistance (*p* = 0.004 and *p* < 0.001). All MMA and MBO isolates were susceptible to amikacin and clarithromycin (day 3) (Table 3 and Figure S1). An ICR was observed in 94.4% (51/54) MAB and 100.0% (7/7) MBO with T28, while no ICR was observed with MMA (Table 4). The susceptibility patterns of other drugs were comparable in the three subspecies. In addition, MABSC isolates were identified as 58.7% (84/143) smooth, 39.2% (56/143) rough, and 2.1% (MAB, n = 3) mixed morphotypes. Of these, 69.6% of the rough morphotypes belonged to MAB, whereas 60.7% of smooth morphotypes were identified as MMA. The rough morphotype was associated with resistance to amikacin, clarithromycin (day 3), and imipenem (*p* < 0.001), whereas the smooth morphotype had a higher MIC for linezolid (*p* = 0.002). The susceptibility patterns, MIC_50_, and MIC_90_ of other drugs were comparable in the two morphotypes (Table 5 and S4).

**Table 4:** Genotypes of the *erm*(41), *rrl*, and *rrs* and antimicrobial resistance of *M. abscessus* complex (MABSC) clinical isolates

Drug	Gene	Genotypic result		Phenotypic resistance ^(a)^/Genotype (N, %)			
		Genotype	N of MABSC isolates	MAB		MBO	MMA
				I	R	R	R
Clarithromycin	*erm*(41) (D3)	T28	125	1/54 (1.9)	17/54 (31.5)	0/7 (0)	0/64 (0)
		C28	18	0	3/18 (16.7) ^(b)^	0	0
	*erm*(41) (D14)	T28	125	0	51/54 (94.4)	7/7 (100.0)	0/64 (0)
		C28	18	0	3/18 (16.7) ^(b)^	0	0
	*rrl*	No mutation	120	0	0/50 (0)	0/6 (0)	0/64 (0)
		A2058C	1	0	1/1 (100.0)	0	0
		A2058C and A2058G	8	0	7/8 (87.5)	0	0
		A2058G	8	0	8/8 (100.0)	0	0
		A2059C	4	1/4 (25.0)	3/4 (75.0)	0	0
		A2058C and A2059C	1	0	1/1 (100.0)	0	0
		A2058G and A2059G	1	0	0	0/1 (0) ^(c)^	0
Amikacin	*rrs*	No mutation	130	1/59 (1.7)	0	0/7 (0)	0/64 (0)
		A1408G	13	7/13 (53.8)	5/13 (38.5)	0	0

^(a)^ Amikacin and clarithromycin resistance was determined on day 3 (D3) of the incubation and inducible clarithromycin resistance (ICR) from *erm*(41) was reexamined on day 14 (D14) of the incubation

^(b)^ Three of the MAB isolates with C28 of the *erm*(41) harbored *rrl* mutations that conferred clarithromycin resistance

^(c)^ One MBO isolate with A2058G and A2059G became clarithromycin resistant after reexamining the MIC plate on day 14 of incubation

Abbreviations: N, number; D3, day 3; D14, day 14; MABSC, *M. abscessus* complex; MAB, *M. abscessus* subsp. *abscessus*; MBO, *M. abscessus* subsp. *bolletii*; MMA, *M. abscessus* subsp. *massiliense*; I, Intermediate; R, Resistant

**Table 5:** Summary of antimicrobial susceptibility of *M. abscessus* complex (MABSC) based on morphotypes

Drugs	N (%) of isolates ^(a)^						*p-value*
	Smooth (N = 84)			Rough (N = 56)			
	S	I	R	S	I	R	
Amikacin	82 (97.6)	2 (2.4)	0	45 (80.4)	6 (10.7)	5 (8.9)	< 0.001 *
Cefoxitin	2 (2.4)	66 (78.6)	16 (19.0)	1 (1.8)	37 (66.1)	18 (32.1)	0.172
Ciprofloxacin	3 (3.6)	9 (10.7)	72 (85.7)	1 (1.8)	3 (5.4)	52 (92.9)	0.504
Clarithromycin (D3)	78 (92.9)	1 (1.2)	5 (6.0)	41 (73.2)	0	15 (26.8)	< 0.001 *
Doxycycline	0	8 (9.5)	76 (90.5)	1 (1.8)	3 (5.4)	52 (92.9)	0.33
Imipenem	1 (1.2)	48 (57.1)	35 (41.7)	1 (1.8)	12 (21.4)	43 (76.8)	< 0.001 *
Linezolid	13 (15.5)	23 (27.4)	48 (57.1)	23 (41.1)	14 (25.0)	19 (33.9)	0.002 *
Moxifloxacin	2 (2.4)	1 (1.2)	81 (96.4)	0	2 (3.6)	54 (96.4)	0.494
TMP/SMX	1 (1.2)	0	83 (98.8)	0	0	56 (100.0)	1
Tobramycin	0	5 (6.0)	79 (94.0)	1 (1.8)	3 (5.4)	52 (92.9)	0.561

* Statistically significant values

^(a)^ Three of the MAB isolates with mixed morphotypes were excluded from the analysis

Abbreviations: N, number; MABSC, *M. abscessus* complex; S, Susceptible; I, Intermediate; R, Resistant; TMP/SMX, Trimethoprim/Sulfamethoxazole.

### Genotypes associated with clarithromycin and amikacin resistance

Mutations in the *erm*(41), *rrl*, and *rrs* genes were investigated (Table 4 and Figure S2). T28 of *erm*(41) was detected in 87.4% (125/143) of MABSC isolates which were MAB (43.2%, 54/125), MBO (5.6%, 7/125), and MMA (51.2%, 64/125). A phenotypic ICR was not observed for three of the MAB and all MMA that had T28. C28 was detected in 25% (18/72) of the MAB that was susceptible to clarithromycin. None of C28 was observed in *erm*(41) of MBO and MMA. For MAB and MBO, the PPV and NPV of molecular detection of T28 conferring an ICR were 95.1% (95% CI, 84.1-98.6%) and 100.0%, respectively. Acquired clarithromycin resistance conferred by *rrl* mutations at positions 2058 and/or 2059 (A2058C, A2058G, A2059C, and A2059G) was detected in 16.1% (23/143) of MABSC isolates (MAB [n = 22] and MBO [n = 1]). Discordant results were observed for one MAB and one MBO isolate that harbor *rrl* mutations but were susceptible to clarithromycin on day 3 of incubation. However, the MBO with A2058G and A2059G became clarithromycin resistant on day 14 of incubation. For amikacin resistance, the *rrs* mutation (A1408G) was detected in 13 MAB isolates recovered from one patient at different time points. These isolates had variable amikacin susceptibilities, which were susceptible (n = 1), intermediate (n = 7), and resistant (n = 5). Overall agreement between the GenoType NTM-DR assay (*rrl* and *rrs)* and a broth microdilution method (intermediate and resistant results considered as R) of clarithromycin and amikacin susceptibility was 98.6% (141/143).

## Discussion

In this study, the MABSC clinical isolates were identified to the subspecies level, and their susceptibilities to 16 antimicrobial agents were determined. In addition, the clinical characteristics of the patients were investigated. In Thailand, this information is still limited, as only a few studies have reported the prevalence and drug susceptibility profiles of MABSC [7, 23]. MABSC is the most common RGM isolated from clinical specimens at our hospital. True MABSC pulmonary infections or NTM-PD can be diagnosed using clinical, radiological, and microbiological criteria [16, 17] and differentiated from specimen contamination [24] or colonization in the respiratory tract. For the diagnosis of extrapulmonary NTM disease, a single positive specimen from a sterile site, body fluid, or tissue biopsy is sufficient [25]. These criteria were applied for the diagnosis of MABSC infection in this study. Most patients with MABSC infections or colonization at our hospital had comorbidities such as immunocompromised status and bronchiectasis, which are associated with NTM infections [13, 26]. In addition, the rate of infections related to medical procedures has increased.

To treat NTM-PD infections, a combination of antimicrobial drugs including oral (azithromycin or clarithromycin, clofazimine, linezolid) and intravenous drugs (amikacin, imipenem or cefoxitin, tigecycline), is recommended and applied for extrapulmonary diseases. However, the optimal duration of treatment has not been universally established [16, 17]. Drug susceptibilities should be determined for clinically significant MABSC isolates. However, MABSC drug susceptibilities may not be the only key factor that influences the clinical outcomes of infected patients. In this study, some patients with favorable outcomes were infected by ICR-MABSC but responded to macrolide-based regimens. However, certain patients who were infected with clarithromycin/amikacin-susceptible MABSC strains experienced treatment failure or death (Table S2). Therefore, other factors, such as drugs used in the regimen, duration of use, severity of the disease, or patient comorbidities [27], could impact the clinical outcomes for the individual patient. In this study, the MABSC subspecies were not statistically associated with clinical outcomes. This could be due to a low number of patients (n = 31) from loss to follow-up, one of the limitations of this study.

The major MABSC subspecies that caused infections and were recovered from clinical specimens in our hospital were MAB followed by MMA. MBO was a relatively rare pathogen, consistent with findings in other countries. In addition, the drug susceptibility patterns of each MABSC subspecies and clinical isolates from various regions and countries can be different [5–9]. Therefore, the MABSC subspecies could be one of the important factors in the selection of an optimal and effective therapeutic regimen for patient management. In this study, MAB was associated with clarithromycin and amikacin resistance. However, the subspeciation analysis of MABSC is not available in most routine diagnostic laboratories. Therefore, the drug susceptibility patterns of the clinical isolates of MABSC without subspeciation are shown in Table 3. Amikacin was still the most effective drug against MABSC, and most MABSC strains were highly resistant to several drugs, including clarithromycin (day 14 of incubation), ciprofloxacin, doxycycline, moxifloxacin, TMP/SMX, and tobramycin, as previously reported [5, 6, 9]. A high intermediate rate was observed for imipenem and cefoxitin, consistent with the findings from previous studies [8, 9, 28]. For drugs without addressed breakpoints, clofazimine and tigecycline could be potential drugs to effectively treat MABSC infections due to their low MIC_50_ and MIC_90_ values, consistent with a study from the United States [8]. On the other hand, amoxicillin/clavulanic acid, cefepime, ceftriaxone, and minocycline may be ineffective in inhibiting the pathogens, so they have not been recommended for MABSC treatment [16, 17].

At present, molecular testing has become an important tool for rapidly detecting the gene mutations associated with clarithromycin and amikacin resistance. In this study, discrepant results between phenotypic and genotypic (GenoType NTM-DR) drug susceptibility results were observed, as previously reported [7, 29]. This indicated that clarithromycin and amikacin resistance might involve other mechanisms apart from those mutations detected in the *erm*(41), *rrl*, and *rrs* genes. In addition, despite the presence of T28, three MAB isolates did not express ICR. These findings suggest a point mutation, especially nonsynonymous or nonsense mutation, leading to a truncated Erm protein [30]. Therefore, sequencing analysis of *erm*(41) should be performed.

For colony morphology, MABSC can switch from a smooth to a rough morphotype, which has been shown to be more invasive and associated with poor clinical outcomes [13, 14]. Our findings show that the rough morphotype was significantly associated with amikacin, clarithromycin, and imipenem resistance, while the smooth morphotype was associated with linezolid resistance. However, the correlations between MABSC morphotype and drug susceptibility are still limited and lacking clarity. A French study reported high MICs of imipenem and cefoxitin in the rough morphotype [31], which was consistent with our findings for imipenem. However, previous studies showed that the MABSC morphotype did not significantly impact antimicrobial susceptibility [32, 33]. Therefore, future genetic analysis of glycolipid (GLP) synthesis or transport genes [34] of MABSC clinical isolates should be performed to confirm their true morphotypes and should study their correlation with drug susceptibility or clinical outcomes.

This study has many strengths, as it is the first study to investigate the clinical and microbiological associations of patients with MABSC infections in Thailand. However, there are some limitations in this study. First, this study included MABSC isolates from both treated and nontreated patients, which could affect the drug susceptibilities from the selection of resistant strains in treated patients. Second, the number of patients and MBO isolates was low. Third, the discrepant results between the phenotypic and genotypic susceptibilities of clarithromycin and amikacin were not further investigated using other methods, such as gene sequencing analysis. Last, this was a single-center study, and most patients came from Central Thailand. None of the patients living with HIV who were particularly vulnerable to NTM infection were recruited for this study. Therefore, these limitations could impact the statistical analysis and might not represent all MABSC isolates from the Thai population and patients living with HIV. Future studies will be conducted with additional patients and MABSC isolates, as well as genetic analysis of the genes associated with MABSC morphotypes and drug susceptibility.

## Conclusions

This study demonstrated the differences in the clinical and microbiological data of patients with MABSC infections caused by the three different subspecies and two morphotypes of MABSC. The findings of this study could be useful for the selection of antimicrobial regimens and the treatment of patients with MABSC infections.

### Electronic supplementary material

Below is the link to the electronic supplementary material.

Supplementary Material 1

## Data Availability

All data generated or analyzed during this study are included in this published article [and its supplementary information files].

## List of Abbreviations

BAL: Bronchoalveolar lavage
IQR: Interquartile range
ICR: Inducible clarithromycin resistance
MABSC: *M. abscessus* complex
MAB: *M. abscessus* subsp. *abscessus*
MBO: *M. abscessus* subsp. *bolletii*
MMA: *M. abscessus* subsp. *massiliense*
MIC: Minimal inhibitory concentration
MIC_50_: Minimal inhibitory concentration required to inhibit the growth of 50% of organisms
MIC_90_: Minimal inhibitory concentration required to inhibit the growth of 90% of organisms
NTM-PD: Nontuberculous mycobacterial pulmonary disease
TB: Tuberculosis
SSTI: Skin and soft-tissue infection
S: Susceptible
I: Intermediate
R: Resistant
TMP/SMX: Trimethoprim/Sulfamethoxazole
N: Number
