# Supplementary material for: Clinical characteristics and drug susceptibility profiles of Mycobacterium abscessus complex infection at a medical school in Thailand
Source: Ann Clin Microbiol Antimicrob. 2023 Sep 21;22:87. doi: 10.1186/s12941-023-00637-4 (PMC10515245; doi:10.1186/s12941-023-00637-4)
Supplement: Supplementary file 1 — Supplementary Material 1 [file 12941_2023_637_MOESM1_ESM.pdf]

Supplementary data

Clinical characteristics and drug susceptibility profiles of *Mycobacterium abscessus* complex infection at a medical school in Thailand

Songkiat Sukmongkolchai*[1], Suthidee Petsong*[2], Nont Oudomying[2,3], Ajala Prommi[4,5], Sunchai Payungporn[5,6], Warat Usawakidwiree[7], Kanphai Wongjarit[2,8], Gompol Suwanpimolkul[8], Kiatichai Faksri[9,10], Chusana Suankratay[8], Suwatchareeporn Rotcheewaphan[#2, 5]

[1]Medical Microbiology, Interdisciplinary and International Program, Graduate School, Chulalongkorn University, Bangkok, Thailand

[2]Department of Microbiology, Faculty of Medicine, Chulalongkorn University, Bangkok, Thailand

[3]Chulalongkorn University International Medical Program (CU-MEDi), Faculty of Medicine, Chulalongkorn University, Bangkok, Thailand

[4]Program in Bioinformatics and Computational Biology, Graduate School, Chulalongkorn University, Bangkok, Thailand

[5]Center of Excellence in Systems Microbiology, Faculty of Medicine, Chulalongkorn University, Bangkok, Thailand

[6]Department of Biochemistry, Faculty of Medicine, Chulalongkorn University, Bangkok, Thailand

[7]Department of Medicine, Faculty of Medicine, Chulalongkorn University, Bangkok, Thailand

[8]Division of Infectious Diseases, Department of Medicine, Faculty of Medicine, Chulalongkorn University, Bangkok, Thailand

[9]Department of Microbiology, Faculty of Medicine, Khon Kaen University, Khon Kaen, Thailand

[10]Research and Diagnostic Center for Emerging Infectious Diseases (RCEID), Khon Kaen University, Khon Kaen, Thailand

Supplementary figures

Drug	Total (n=143)	Subspecies: MAB (n=72)	Subspecies: MBO (n=7)	Subspecies: MMA (n=64)	Morphotypes: Smooth (n=84)	Morphotypes: Rough (n=56)
Amikacin	3.5	6.9	0	0	0	8.9
Cefoxitin	23.8	33.3	14.3	14.1	19.0	32.1
Ciprofloxacin	88.8	91.7	85.7	85.9	85.7	92.9
Clarithromycin (D3)	14.0	27.8	0	0	6.0	26.8
Doxycycline	91.6	88.9	100.0	93.8	90.5	92.9
Imipenem	56.6	63.9	57.1	48.4	41.7	76.8
Linezolid	48.3	47.2	14.3	53.1	57.1	33.9
Moxifloxacin	96.5	94.4	100.0	98.4	96.4	96.4
TMP/SMX	99.3	100.0	100.0	98.4	98.8	100.0
Tobramycin	93.7	88.9	100.0	98.4	94.0	92.9

100%
80%
60%
40%
20%
0%

Figure S1. Resistant *M. abscessus* complex (MABSC) clinical isolates. Numbers shown are the percentage of resistant MABSC tested by a broth microdilution method and interpreted according to the CLSI guideline [1]. Three MAB isolates with mixed morphotypes were excluded from the morphotype analysis.

Abbreviations: n, number; MABSC, *M. abscessus* complex; MAB, *M. abscessus* subsp. *abscessus*; MBO, *M. abscessus* subsp. *bolletii*; MMA, *M. abscessus* subsp. *massiliense*; TMP/SMX, Trimethoprim/Sulfamethoxazole.

A) Clarithromycin

M. abscessus* subsp. *abscessus

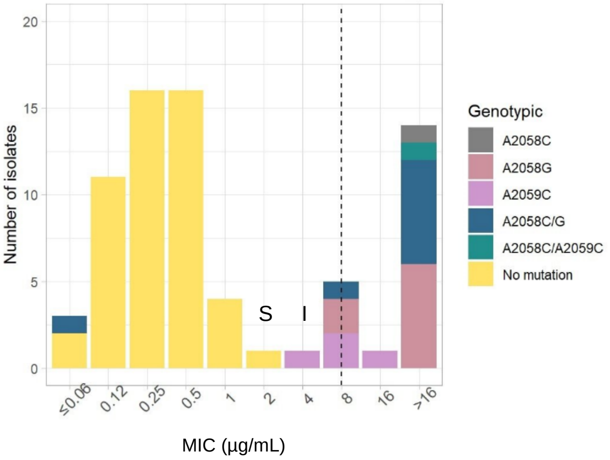

M. abscessus* subsp. *massiliense

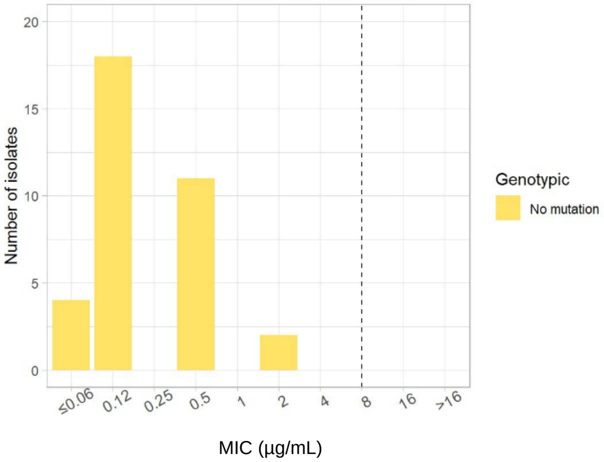

M. abscessus* subsp. *bolletii

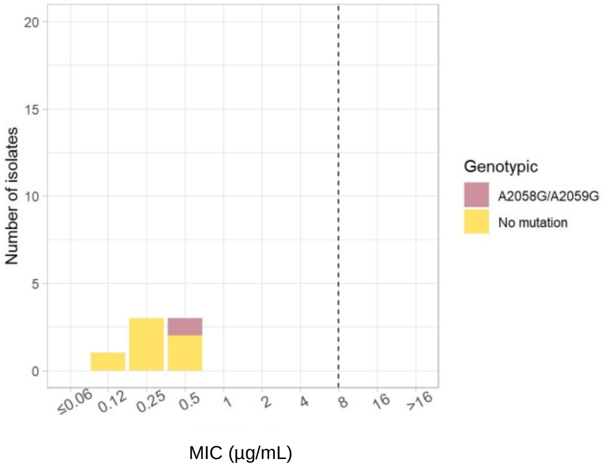

B) Amikacin

M. abscessus* subsp. *abscessus

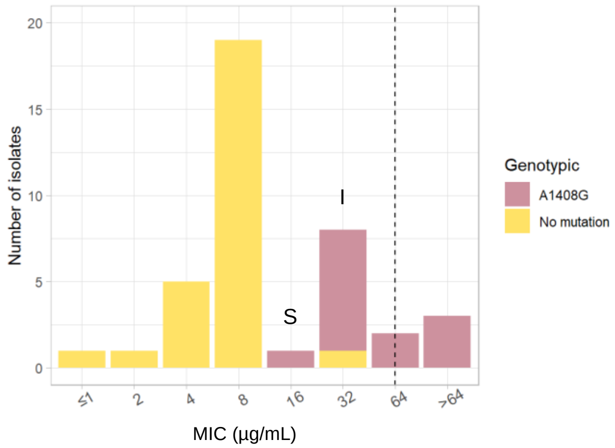

M. abscessus* subsp. *massiliense

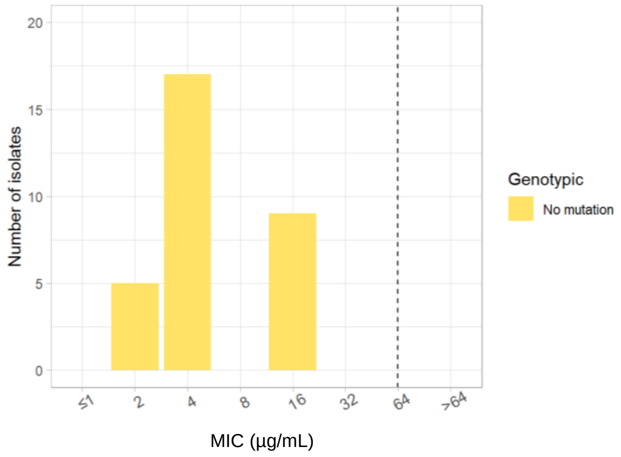

M. abscessus* subsp. *bolletii

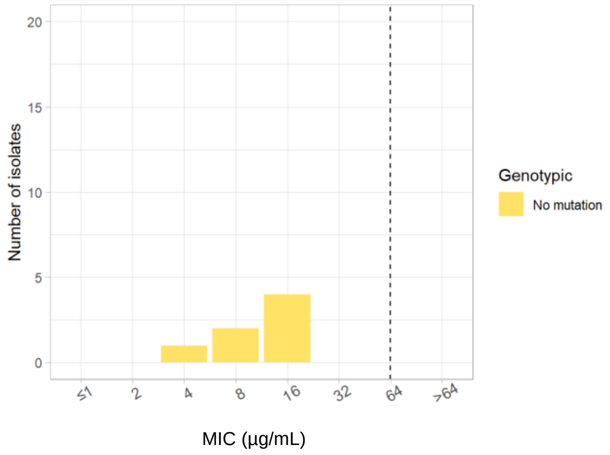

Figure S2. Distributions of MIC values (day 3) and resistance-conferring mutations of clarithromycin (A) and amikacin (B) of the MABSC subspecies. MIC values with the dashed lines and higher MICs are interpreted to be resistant.

Abbreviations: MIC, Minimal inhibitory concentration; S, Susceptible; I, Intermediate.

Supplementary tables

Table S1. Antimicrobial concentration ranges and MIC breakpoints for drug susceptibility testing

Antimicrobial agents (RAPMYCOI plate)	Range of concentrations evaluated (µg/mL) [(a)]	CLSI breakpoints (µg/mL)		
		S	I	R
Amikacin (IV)	1 to 64	≤16	32	≥64
Cefoxitin	4 to 128	≤16	32 - 64	≥128
Ciprofloxacin	0.12 to 4	≤1	2	≥4
Clarithromycin	0.06 to 16	≤2	4	≥8
Doxycycline	0.12 to 16	≤1	2 - 4	≥8
Imipenem	2 to 64	≤4	8 - 16	≥32
Linezolid	1 to 32	≤8	16	≥32
Moxifloxacin	0.25 to 8	≤1	2	≥4
Trimethoprim/sulfamethoxazole	0.25/4.75 to 8/152	≤2/38	-	≥4/76
Tobramycin	1 to 16	≤2	4	≥8
Amoxicillin/clavulanic acid	2/1 to 64/32	-	-	-
Cefepime	1 to 32	-	-	-
Ceftriaxone	4 to 64	-	-	-
Minocycline	1 to 8	-	-	-
Tigecycline	0.015 to 4	-	-	-
Clofazimine [(b)]	0.03 to 4	-	-	-

[(a)] For drugs without addressed breakpoints, MIC values were reported as the CLSI guideline recommendation [1, 2].

[(b)] Clofazimine is not included in the Sensititre Myco RAPMYCOI plate.

Abbreviations: MIC, Minimal inhibitory concentration; S, Susceptible; I, Intermediate; R, Resistant.

Results

Table S2. Clinical and microbiological characteristics of patients with MABSC pulmonary or extrapulmonary diseases (Patients 1-29)

Patient [(a)]	Sex	Age	Clinical specimens with positive MABSC (N)	Comorbidities related to NTM infections	NTM diseases	Clinical outcome	MABSC subspecies	Clarithromycin susceptibility [(b)]		Amikacin susceptibility [(b)]	*erm*(41) (Position 28)	*rrl* mutation	*rrs* mutation
								D3	D14				
1	F	62	Sputum (4)	No underlying disease	Disseminated disease	Clinical cure	MAB	S	R	S	T28	Not detected	Not detected
2	F	50	Pus/Wound aspirate (2)	Procedure-related	SSTI	Clinical cure	MMA	S	S	S	T28	Not detected	Not detected
3	M	26	Tissue biopsy (2), Sputum (1), Blood (2)	Acquired immunodeficiency	Disseminated disease	Treatment failure	MBO	S	R	S	T28	Not detected	Not detected
4	F	43	Pus/Wound aspirate (2)	No underlying disease	SSTI	Clinical cure	MMA	S	S	S	T28	Not detected	Not detected
5	M	67	Sputum (5)	Structural lung disease	Pulmonary disease	Treatment failure	MAB	S	R	I	T28	Not detected	Not detected
6	F	38	Sputum (13)	Structural lung disease	Pulmonary disease	Treatment failure	MAB	R	R	R	T28	Detected	Detected
7	M	68	Tissue biopsy (4) [abdominal mass, pancreas, lymph nodes)	Acquired immunodeficiency	Disseminated disease	Death	MAB	S	R	S	T28	Not detected	Not detected
8	F	69	Tissue biopsy (1)	No underlying disease	Disseminated disease	Clinical cure	MAB	S	R	S	T28	Not detected	Not detected
9	F	48	Sputum (8)	Acquired immunodeficiency	Pulmonary disease	Treatment failure	MAB	R	R	S	T28	Detected	Not detected
10	F	64	Sputum (2)	Structural lung disease	Pulmonary disease	Treatment failure	MAB	S	S	S	C28	Not detected	Not detected
11	F	77	Sputum (3)	Structural lung disease	Pulmonary disease	Treatment failure	MMA	S	S	S	T28	Not detected	Not detected
12 [(c)]	M	52	Tissue biopsy (1)	Acquired immunodeficiency	Disseminated disease	Clinical cure	MBO	S	R	S	T28	Not detected	Not detected
13	F	74	Sputum (1)	Structural lung disease	Pulmonary disease	Death	MMA	S	S	S	T28	Not detected	Not detected
14	M	70	BAL (2)	Acquired immunodeficiency	Pulmonary disease	Clinical cure	MMA	S	S	S	T28	Not detected	Not detected
15	F	71	Tissue biopsy (2)	Procedure-related	SSTI	Clinical cure	MMA	S	S	S	T28	Not detected	Not detected
16	F	71	Sputum (2)	Structural lung disease	Pulmonary disease	Clinical cure	MAB	S	S	S	C28	Not detected	Not detected
17	M	70	Tissue biopsy (1)	Acquired immunodeficiency	SSTI	Treatment failure	MMA	S	S	S	T28	Not detected	Not detected
18	F	20	Tissue biopsy (1)	No underlying disease	SSTI	Clinical cure	MMA	S	S	S	T28	Not detected	Not detected
19	F	66	Tissue biopsy (1)	No underlying disease	SSTI	Clinical cure	MAB	S	R	S	T28	Not detected	Not detected
20	M	47	Sputum (1)	No underlying disease	Pulmonary disease	Clinical cure	MMA	S	S	S	T28	Not detected	Not detected

Table S2 (continued). Clinical and microbiological characteristics of patients with MABSC pulmonary or extrapulmonary diseases (Patients 1-29)

Patient [(a)]	Sex	Age	Clinical specimens with positive MABSC (N)	Comorbidities related to NTM infections	NTM diseases	Clinical outcome	MABSC subspecies	Clarithromycin susceptibility [(b)]		Amikacin susceptibility [(b)]	*erm*(41) (Position 28)	*rrl* mutation	*rrs* mutation
								D3	D14				
21	F	40	Tissue biopsy (1)	Acquired immunodeficiency	Lymphadenitis	Clinical cure	MAB	S	R	S	T28	Not detected	Not detected
22	F	50	Tissue biopsy (1)	No underlying disease	SSTI	Clinical cure	MAB	S	R	S	T28	Not detected	Not detected
23	M	68	Tissue biopsy (1)	Acquired immunodeficiency	Disseminated disease	Clinical cure	MMA	S	S	S	T28	Not detected	Not detected
24	F	45	BAL (1)	Structural lung disease	Pulmonary disease	Culture conversion	MMA	S	S	S	T28	Not detected	Not detected
25	F	53	Tissue biopsy (1)	Acquired immunodeficiency	Disseminated disease	Clinical cure	MMA	S	S	S	T28	Not detected	Not detected
26	M	51	Pus/Wound aspirate (1)	Procedure-related	Lymphadenitis	Clinical cure	MMA	S	S	S	T28	Not detected	Not detected
27	M	70	Pus/Wound aspirate (1)	Procedure-related	SSTI	Death from other conditions	MMA	S	S	S	T28	Not detected	Not detected
28	M	69	Sputum (3)	Acquired immunodeficiency	Pulmonary disease	Death	MAB	S	S	S	C28	Not detected	Not detected
29	F	12	Tissue biopsy (1)	No underlying disease	Lymphadenitis	Clinical cure	MAB	S	R	S	T28	Not detected	Not detected

[(a)] Patients (N=29) were diagnosed with NTM-PD or extrapulmonary diseases with known clinical outcomes and treatment regimens.

[(b)] For patients with multiple MABSC isolates recovered at different time points, clarithromycin and amikacin susceptibilities of the MABSC isolate with the highest MIC value were reported.

[(c)] Patient 12 had MBO disseminated infection which was diagnosed by positive MBO isolated from the lymph node and the radiological study with evidence of dissemination to the bone, liver, and spleen.

Abbreviations: F, Female; M, Male; N, Number; MAB, *M. abscessus* subsp. *abscessus*; MBO, *M. abscessus* subsp. *bolletii*; MMA, *M. abscessus* subsp. *massiliense*; SSTI, Skin and soft-tissue infection; BAL, Bronchoalveolar lavage.

Table S3. Summary of antimicrobial susceptibility of *M. abscessus* complex (MABSC) based on subspecies

Drugs	MIC (µg/mL)											
	Total (N=143)			MAB (N=72)			MBO (N=7)			MMA (N=64)		
	Range	MIC_{50}	MIC_{90}	Range	MIC_{50}	MIC_{90}	Range	MIC_{50}	MIC_{90}	Range	MIC_{50}	MIC_{90}
Amikacin	≤1 to >64	8	16	≤1 to >64	16	32	4 to 16	16	16	2 to 16	8	16
Cefoxitin	16 to >128	64	>128	16 to >128	64	>128	32 to 128	64	128	16 to >128	64	128
Ciprofloxacin	0.5 to >4	>4	>4	1 to >4	>4	>4	2 to >4	>4	>4	0.5 to >4	>4	>4
Clarithromycin (D3)	≤0.06 to >16	0.25	16	≤0.06 to >16	0.5	>16	0.12 to 0.5	0.25	0.5	≤0.06 to 2	0.25	0.5
Doxycycline	1 to >16	>16	>16	1 to >16	>16	>16	8 to >16	>16	>16	2 to >16	>16	>16
Imipenem	≤2 to >64	32	>64	≤2 to >64	32	>64	16 to >64	32	>64	≤2 to >64	16	>64
Linezolid	≤1 to >32	16	>32	2 to >32	16	>32	4 to >32	8	>32	≤1 to >32	32	>32
Moxifloxacin	1 to >8	>8	>8	1 to >8	8	>8	4 to >8	8	>8	2 to >8	>8	>8
TMP/SMX	1/19 to >8/152	>8/152	>8/152	4/76 to >8/152	>8/152	>8/152	>8/152	>8/152	>8/152	1/19 to >8/152	>8/152	>8/152
Tobramycin	≤1 to >16	8	>16	≤1 to >16	8	16	8 to >16	8	>16	4 to >16	8	>16
Amoxicillin/clavulanic acid [(a)]	32/16 to >64/32	>64/32	>64/32	32/16 to >64/32	>64/32	>64/32	>64/32	>64/32	>64/32	32/16 to >64/32	>64/32	>64/32
Cefepime [(a)]	16 to >32	>32	>32	16 to >32	>32	>32	>32	>32	>32	>32	>32	>32
Ceftriaxone [(a)]	32 to >64	>64	>64	32 to >64	>64	>64	>64	>64	>64	>64	>64	>64
Clofazimine [(a)] [(b)]	0.12 to 0.5	0.25	0.5	0.12 to 0.5	0.25	0.5	0.12 to 0.5	0.25	0.5	0.12 to 0.5	0.25	0.5
Minocycline [(a)]	4 to >8	>8	>8	4 to >8	>8	>8	>8	>8	>8	4 to >8	>8	>8
Tigecycline [(a)]	0.25 to >4	1	2	0.25 to >4	1	2	0.25 to >4	0.5	>4	0.25 to >4	1	2

[(a)] Drugs without addressed CLSI breakpoints.

[(b)] MIC data for clofazimine could not be determined for one of the MMA isolates (N=63).

Abbreviations: N, number; MIC, Minimal inhibitory concentration; MIC_{50}, Minimal inhibitory concentration required to inhibit the growth of 50% of organisms; MIC_{90}, Minimal inhibitory concentration required to inhibit the growth of 90% of organisms; MABSC, *M. abscessus* complex; MAB, *M. abscessus* subsp. *abscessus*; MBO, *M. abscessus* subsp. *bolletii*; MMA, *M. abscessus* subsp. *massiliense*; TMP/SMX, Trimethoprim/Sulfamethoxazole.

Table S4. Summary of antimicrobial susceptibility of *M. abscessus* complex (MABSC) based on morphotypes

Drugs	MIC (µg/mL)					
	Smooth (N=84)			Rough (N=56)		
	Range	MIC_{50}	MIC_{90}	Range	MIC_{50}	MIC_{90}
Amikacin	2 to 32	8	16	≤1 to <64	8	32
Cefoxitin	16 to >128	64	128	16 to >128	64	>128
Ciprofloxacin	0.5 to > 4	>4	>4	1 to >4	>4	>4
Clarithromycin (D3)	≤0.06 to >16	0.25	1	≤0.06 to >16	0.25	>16
Doxycycline	2 to >16	>16	>16	1 to >16	>16	>16
Imipenem	≤2 to >64	16	>64	≤2 to >64	>64	>64
Linezolid	2 to >32	32	>32	≤1 to >32	16	>32
Moxifloxacin	1 to >8	>8	>8	2 to >8	8	>8
TMP/SMX	1 to >8	>8	>8	4 to >8	>8	>8
Tobramycin	4 to >16	8	>16	≤1 to >16	8	>16
Amoxicillin/clavulanic acid [(a)]	32 to >64	>64	>64	32 to >64	>64	>64
Cefepime [(a)]	32 to >32	>32	>32	16 to >32	>32	>32
Ceftriaxone [(a)]	32 to >64	>64	>64	32 to >64	>64	>64
Clofazimine [(a)] [(b)]	0.12 to 0.5	0.25	0.5	0.12 to 0.5	0.25	0.5
Minocycline [(a)]	4 to >8	>8	>8	4 to >8	>8	>8
Tigecycline [(a)]	0.25 to >4	1	2	0.25 to >4	1	2

[(a)] Drugs without addressed CLSI breakpoints.

[(b)] MIC data for clofazimine could not be determined for one of the smooth MMA isolates (N=83).

Abbreviations: N, number; MIC, Minimal inhibitory concentration; MIC_{50}, Minimal inhibitory concentration required to inhibit the growth of 50% of organisms; MIC_{90}, Minimal inhibitory concentration required to inhibit the growth of 90% of organisms; MABSC, *M. abscessus* complex; TMP/SMX, Trimethoprim/Sulfamethoxazole.

References

[1] CLSI. Performance Standards for Susceptibility Testing of Mycobacteria, *Nocardia* spp., and Other Aerobic Actinomycetes. 2nd edition. CLSI supplement M24S.: Clinical and Laboratory Standards Institute; 2023.

[2] CLSI. Susceptibility Testing of Mycobacteria, *Nocardia* spp., and Other Aerobic Actinomycetes, 3rd ed. CLSI document M24. Wayne, PA: Clinical and Laboratory Standards Institute; 2018.
